# Near-Complete Genome Sequence of a Novel Single-Stranded RNA Virus Discovered in Indoor Air

**DOI:** 10.1128/genomeA.00198-18

**Published:** 2018-03-22

**Authors:** Karyna Rosario, Noah Fierer, Mya Breitbart

**Affiliations:** a College of Marine Science, University of South Florida, Saint Petersburg, Florida, USA; b Department of Ecology and Evolutionary Biology, University of Colorado, Boulder, Colorado, USA; c Cooperative Institute for Research in Environmental Sciences, University of Colorado, Boulder, Colorado, USA

## Abstract

Viral metagenomic analysis of heating, ventilation, and air conditioning (HVAC) filters recovered the near-complete genome sequence of a novel virus, named HVAC-associated RNA virus 1 (HVAC-RV1). The HVAC-RV1 genome is most similar to those of picorna-like viruses identified in arthropods but encodes a small domain observed only in negative-sense single-stranded RNA viruses.

## GENOME ANNOUNCEMENT

A novel RNA virus was detected through metagenomic sequencing of partially purified virus particles from heating, ventilation, and air conditioning (HVAC) filters used as passive indoor air samplers in university dormitory rooms (1). This virus, named HVAC-associated RNA virus 1 (HVAC-RV1), shared low sequence identity with known positive-sense single-stranded RNA (ssRNA) viruses. While HVAC-RV1 was originally identified in a male-occupied dorm room, metagenomic sequences with significant similarities to HVAC-RV1 were identified in 50% (*n* = 12) of the dormitory rooms sampled. The divergent HVAC-RV1 sequence, combined with its prevalence, prompted further investigation and confirmation of this novel viral genome.

A 9.8-kb contig sequence representing HVAC-RV1 was retrieved after assembling quality-filtered metagenomic reads (raw reads available under Sequence Read Archive [SRA] accession number SRR5853132) using SPAdes version 3.6 (2). Based on BLASTx comparisons against the GenBank nonredundant (nr) database, the contig sequence was most similar to Milolii virus, an RNA viral genome identified in ghost ants (3). However, the HVAC-RV1 contig sequence was smaller than the Milolii virus genome (∼10.5 kb) and contained a major open reading frame (ORF) that was truncated (i.e., no stop codon). Therefore, the complete coding region of the assembled HVAC-RV1 genome sequence was verified through PCR and Sanger sequencing to eliminate potential assembly errors. In addition, the 3ʹ end of the assembled sequence was extended using an oligo(dT) adapter primer provided with the rapid amplification of cDNA ends (RACE) system (Invitrogen) (4), indicating that the genome is polyadenylated.

The verified near-complete HVAC-RV1 genome sequence is ∼10.6 kb in size and contains two major nonoverlapping ORFs flanked by 5ʹ and 3ʹ untranslated regions. Searches against the NCBI Conserved Domain Database (5) (E value < 0.001) revealed that ORF1, which is ∼6.4 kb in length, contains RNA helicase (pfam00910), trypsin-like serine peptidase (pfam13365), and RNA-dependent RNA polymerase (pfam00680) conserved domains. This domain organization within polyproteins is reminiscent of picorna-like viruses (6, 7). However, unlike known picorna-like nonsegmented viral genomes, HVAC-RV1 encodes a second major ORF immediately downstream from ORF1 that contains both nonstructural and structural domains. ORF2, which is ∼3.4 kb in length, contains domains with significant similarities to bunyavirus nonstructural NS-S (pfam03231) and calicivirus coat (pfam00915) proteins. The presence of a domain similar to the bunyavirus NS-S protein family is surprising, since this domain has only been reported from negative-sense ssRNA viruses from the order Bunyavirales (8).

BLASTp comparisons of HVAC-RV1 ORF1 and ORF2 hypothetical protein sequences revealed limited similarities (< 40% identity) to polyproteins from the Milolii virus and other unclassified arthropod-associated viruses whose picorna-like genomes are predicted to encode a single polyprotein. Therefore, HVAC-RV1 contains a unique genome organization and further illustrates that current taxonomic classification schemes need to be reassessed to accommodate expanding RNA viral diversity (9).

### Accession number(s).

The near-complete genome sequence of the HVAC-associated RNA virus 1 has been deposited in GenBank under the accession number MG775312.
